# A novel framework for horizontal and vertical data integration in cancer studies with application to survival time prediction models

**DOI:** 10.1186/s13062-019-0249-6

**Published:** 2019-11-21

**Authors:** Iliyan Mihaylov, Maciej Kańduła, Milko Krachunov, Dimitar Vassilev

**Affiliations:** 1 Faculty of Mathematics and Informatics, Sofia University, “St. Kliment Ohridski”, 5 James Bourchier Blvd., Sofia, 1164 Bulgaria; 2 Department of Biotechnology, Boku University, Vienna, 1180 Austria; 3 Institute for Machine Learning, Johannes Kepler University, Linz, 4040 Austria

**Keywords:** Breast cancer, Neuroblastoma, Semantic data integration, Machine learning, Survival time prediction

## Abstract

**Background:**

Recently high-throughput technologies have been massively used alongside clinical tests to study various types of cancer. Data generated in such large-scale studies are heterogeneous, of different types and formats. With lack of effective integration strategies novel models are necessary for efficient and operative data integration, where both clinical and molecular information can be effectively joined for storage, access and ease of use. Such models, combined with machine learning methods for accurate prediction of survival time in cancer studies, can yield novel insights into disease development and lead to precise personalized therapies.

**Results:**

We developed an approach for intelligent data integration of two cancer datasets (breast cancer and neuroblastoma) − provided in the CAMDA 2018 ‘Cancer Data Integration Challenge’, and compared models for prediction of survival time. We developed a novel semantic network-based data integration framework that utilizes NoSQL databases, where we combined clinical and expression profile data, using both raw data records and external knowledge sources. Utilizing the integrated data we introduced Tumor Integrated Clinical Feature (TICF) − a new feature for accurate prediction of patient survival time. Finally, we applied and validated several machine learning models for survival time prediction.

**Conclusion:**

We developed a framework for semantic integration of clinical and omics data that can borrow information across multiple cancer studies. By linking data with external domain knowledge sources our approach facilitates enrichment of the studied data by discovery of internal relations. The proposed and validated machine learning models for survival time prediction yielded accurate results.

**Reviewers:**

This article was reviewed by Eran Elhaik, Wenzhong Xiao and Carlos Loucera.

## Background

In the last decade, high-throughput technologies have been massively used alongside clinical tests to study various diseases in order to decipher the underlying biological mechanisms and devise novel therapeutic strategies. The generated high-throughput data often correspond to measurements of different biological entities (e.g., transcripts, proteins), represent various views on the same entity (e.g., genetic, epigenetic) and are created through different technologies (e.g., microarrays, RNA-Sequencing). The data are heterogeneous, of different types and formats. There is an obvious necessity to integrate the data, in order to store, access, relate, analyse and mine them easily.

Data integration is understood as a mean to combining data from different sources, creating a unified view and improving their accessibility to a potential user [1–3]. Data integration and biomedical analyses are separate disciplines and have evolved in relative isolation. There is a general agreement that uniting both these disciplines in order to develop more sustainable methods for analysis is necessary [4, 5]. Data integration fundamentally involves querying across different data sources. These data sources could be, but are not limited to, separate relational databases or semi-structured data sources distributed across a network. Data integration facilitates dividing the whole data space into two major dimensions, referring to where data or knowledge about metadata reside and to the representation of data and data models. Biomedical experiments take advantage of a vast number of different analytical methods that facilitate mining relevant data from the dispersed information. Some of the most frequent experiments are related to gene expression profiling, clinical data analytics [6], rational drug design [7, 8], which attempt to use all available biological and clinical knowledge to make informed development decisions. Moreover, machine learning-based approaches for finding and highlighting the useful knowledge in the vast space of abundant and heterogeneous data are applied for improving these analytics. Metadata, in particular, are gaining importance, being captured explicitly or inferred with help of machine learning models. Some examples include the use of machine learning methods for the inference of data structure, data distribution, and common value patterns.

**Fig. 1:**
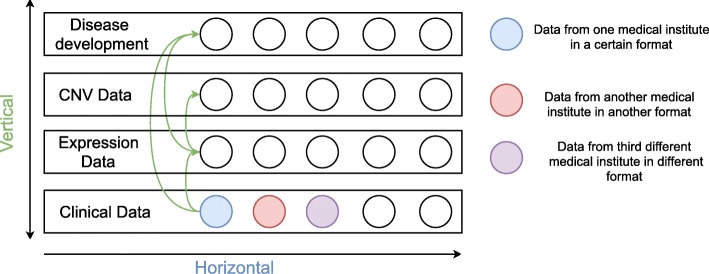
Horizontal and vertical data integration. Green arrows show relations between the data types (clinical, expression, CNV and disease development, i.e. cancer progression). Horizontal integration is between patients, where the data can originate from, e.g., different institutes, but covers the same type of data. The vertical integration is applied to combine the different data types

The heterogeneity of data makes any integrative analysis highly challenging. Data generated with different technologies include different sets of attributes. Where data are highly heterogeneous and weakly related two interconnected integrative approaches are applied: horizontal and vertical integration (Fig. 1). The horizontal data integration unites information of the same type, but from different data sources and, potentially, in different formats. It facilitates uniting heterogeneous data, like clinical information, from many different sources in one data model. The vertical data integration, on the other hand, means relating different analyses and knowledge across multiple types of data, helping to manage links between the patient’s gene expression, clinical information, available chemical knowledge, and existing ontologies. Most existing approaches for data integration focus on one type of data or one disease and cannot facilitate cross-type or -disease integration [9, 10].

## Related work

In this work horizontal integration is considered to be a management approach in which the raw data (patients, clinical records, expression profiles, etc.) can be “owned” and managed by one network. Usually, each type of raw data can define different semantics for common management purposes. In contrast, vertical integration semantically combines the attributes of each separate type of data that are related to one another. Additional information, in particular for the molecular data, can be found in external domain knowledge sources. With this newly added information the missing parts of the studied data can be filled in. In this way relations between attributes of the different records can be learnt. Currently, there are many established algorithms that address single-track data analysis [7, 8, 11, 12], and some recent successful approaches to integrative exploration [13]. These, however, usually only focus on one of the integration applications, either horizontal or vertical, underutilizing the entireness of the available information and the latent relations. We propose a novel framework that employs both these integration views. We show its value on a first example application to machine learning-based survival time prediction.

## Novel model

In this study we combine data from neuroblastoma (NB) and breast cancer (BC). Via our data integration approach whole datasets are joined, but the semantic integrity of the data is kept and enriched. Through combining data from multiple cancers in this way we create a network of data where entities, like proteins, clinical features and expression features, are linked with each other [14]. Data can be often represented as networks, where nodes indicate biologically relevant entities (typically genes or proteins) and edges represent relationships between these entities (e.g., regulation, interaction). In our generated network, nodes represent patients and edges represent similarities between the patients’ profiles, consisting of clinical data, expression profiles and copy number information. Such network can be used to group similar patients and to associate these groups with distinct features [15]. The main challenges here are: (1) building an appropriate linked data network, discovering a semi-structure of the data model [16] and mapping assertions by the applied model for data integration [17]; and (2) data cleaning, combined into a formal workflow for data integration.

We focus on two aspects of data integration: horizontal and vertical. As explained, horizontal data integration means combining data within the same data source. In the datasets analysed here, the data sources are, specifically: clinical information, expression profiles and copy number data. Each type of data is measured by a different technology and potentially available in various data formats. As an example, we treat clinical data from two cancers as one data source, or one entity, even if it is in different formats. These entities are, however, semantically similar. Vertical data integration, on the other hand, is applied to creating relations between all horizontally integrated objects. This vertical data integration provides a connection between all different types of entities. This connection covers relations between patients through clinical information, expression and copy number profiles. Based on these relations we can easily detect all patients closely related to each other by, for instance, protein mutations, diagnosis and/or therapy.

Different databases are required for horizontal and for vertical data integration because each of these approaches address different aspects of the integration problem. Horizontal data integration deals with unstructured and heterogeneous data. Thus, we use a document-based database (such as MongoDB), which can handle different data types and formats. For vertical data integration a graph-based database is applied, as it is suitable for representing relations − crucial in this case. In this study, all relations are established between existing records for each entity, and represented by a semi-structure.

Data integration model with a NoSQL database can potentially unite medical studies data, alternatively to the most frequently used statistical/machine learning methods. Most of the NoSQL database systems share common characteristics, supporting the scalability, availability, flexibility and ensuring fast access times for storage, data retrieval and analysis [18, 19]. Very often when applying cluster analysis methods for grouping or joining data issues occur − mainly with outliers, small classes, and mostly with data dynamically changing relatedness. To overcome these problems a NoSQL database integration model can be applied. Further we extend the potential of the model by using multiple datasets, regardless of the level of heterogeneity, formats, types of data, etc. − all very relevant in cancer studies [20].

Our integrative framework facilitates direct analyses of the data. We first focus on a specific clinically relevant application: modeling and prediction of the survival time of cancer patients. This consists of applying both conventional classification methods and machine learning algorithms. Via data integration a new integrated and universal, i.e. applicable to both cancers, feature for survival time prediction is introduced. This feature is built from three clinical features which are most related to survivability. This integrated feature, further, provides a connection to the newly developed linked data network. This feature is used, in conventional classification k-neighbours method, to find patients that are related most closely to the studied one. After that, via the linked data we find other patients who may not have the new integrative feature but are still related by different types of data, like gene expression or CNV. Machine learning models, based on support vector and decision tree regression, are then used for survival time prediction and cross validation.

## Material and methods

Our multilayer model for data integration consists of linked and internal networks built for both of the studied types of cancer: neuroblastoma and breast cancer. Both of these cancers include several types of data for each patient, such as expression data, copy number data and corresponding clinical information. In order to find common mutated proteins and to provide common therapies, we gain insight about the clinical outcome by detecting relations between these multiple types of data. By using such built relations we can find patients closest to the studied patient of interest, based on semantic similarity of diagnosis, applied therapy and gene expression profile. With this data integration model, which contains linked and relevant knowledge, we can build a specific network for each studied patient.

Modeling relations between molecular data sources and the linked information (clinical data, molecular data sources, patient records, etc.) is a crucial aspect of data integration in our study. In this regard two basic approaches have been proposed. The first approach, called here ‘internal data network’, requires data to be expressed in terms of internal relations. These relations can be found directly in the raw data. The second approach, called ‘linked data network’, requires the data to be “enriched” by using external domain knowledge sources [21].

Specifically, molecular data can be linked with external domain knowledge sources, like pathway and protein databases, by a general approach known as Linked Data schema. Linked Data is a method of publishing structured data so that it can be interlinked and become more informative through semantic queries. It is built upon standard Web technologies such as Hypertext Transfer Protocol (HTTP, [1]), Representational State Transfer (RESTful) and Uniform Resource Identifiers (URIs) and extends them to share information in a way that can be read automatically by computers, mostly via RESTful APIs [22].

The structure of Linked Data is based on a set of principles and standard recommendations created by the W3C. Single data points are identified with HTTP [1] URIs. Similar to how a web page can be retrieved by resolving its HTTP URI (e.g., ‘http://en.wikipedia.org/wiki/Presenilin’), data including a single entity in the Linked Data space can be retrieved by resolving its HTTP URI (e.g., ‘http://dbpedia.org/resource/Presenilin’). In order to “impute” missing parts of the integrated data, like protein annotations, protein relationships, mutations, finding hidden protein motifs, etc., it is necessary to use Linked Data from different domain knowledge sources, like UniProt, Ensembl, GO databases [23, 24]. This is defined as another network layer over the already built one in the data integration step. In Linked Data space all entities are interlinked. This results in one large overarching network where objects are interrelated. The challenge here is to apply this network to finding more complete and reliable information for each of the studied patients, as well as to be able to use this information for survival time prediction modeling.

### Data description

**Fig. 2:**
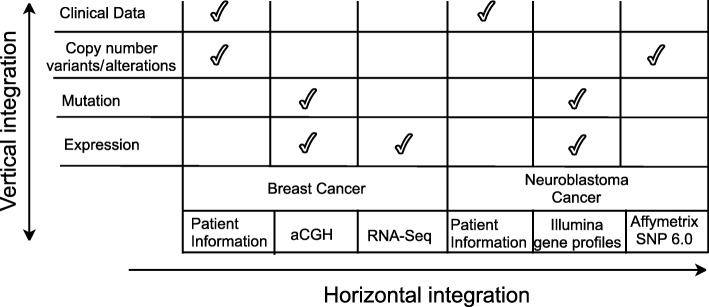
Raw data specific to the investigated datasets. Along the horizontal arrow multiple different data types for a particular patient are shown. Along the vertical arrow integrated types of data related to the studied cancers and linked to a certain patient are given

Two datasets − neuroblastoma (NB) [12] and breast cancer (BC) [25], are used in this study. Data were provided by the CAMDA 2018 challenge [26]. Similar type of information is provided by different sources in different formats. The neuroblastoma dataset contains RNA-Seq gene expression profiles of 498 patients as well as Agilent microarray expression and aCGH copy number data for a matched subset of 145 patients each, and corresponding clinical information. The breast cancer set contains profiles for microarray expression and CNV copy number data, and clinical information (survival time, multiple prognostic markers, therapy data) for about 2,000 patients. The types of data and information sources are shown in Fig. 2. We integrate all data both horizontally and vertically.

### Data preprocessing

For initial data preprocessing we developed a programming module in Python (version 3.7) with library scikit-learn [27, 28] for reading in the raw files. The module automatically discovers the delimiter which separates each attribute in the raw data files. Each file has a header with rows, containing specific information about the file, the technology applied for generation of this file, types and number of attributes, and references to other files (clinical data files have reference to expression files via file ID). Our programming module reads this information in and uses it to create a so-called semi-structure. This semi-structure contains attributes which exist in each type of data. Data types include: clinical information, expression and copy number profiles (Fig. 1). This module is used to build a semi-structure repeatedly and iteratively, record by record. Each record is built from fields/attributes (all values from one record). For each record we store aggregated information for all fields in one data structure, which contains two parameters − field name and count of repeated fields [29]. When new fields are added to the data semi-structure they are imported into our database. The database consists of two layers − first: non relational document-based database; and second: graph-based database. This way the workflow is completed and raw data are integrated into the database as a data semi-structure. These fields − in each record, represent a small set of all fields/attributes. In the document-based database we apply a restriction (called ‘data schema’) based on the generated semi-structure. The applied data schema over each record for each type of data joins data in different formats and from different sources. For each type of data this data schema always contains ID and the Sample ID (representing the name of the subject, as provided in the clinical information).

### Data integration

Utilizing the semi-structure, heterogeneous data are integrated into one database, where the final goal is to create a network of relations between all types of data. In these networks, nodes represent patients and edges represent similarities between patient profiles. The similarity means that two patients are related to each other by multiple proteins, based on expression profiles and copy number changes. These networks of relations facilitate grouping of the patients. Patient groups can then be associated with distinct clinical outcome.

**Fig. 3:**
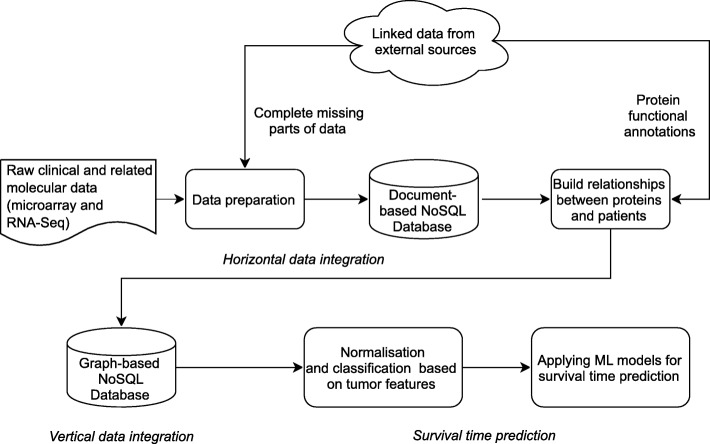
Workflow of data integration of the independent datasets, performed within our framework. In data preparation phase we transform and store the raw data of different formats in a document database, performing horizontal data integration per data type. We generate relations between the data based on the available raw patient datasets, including clinical information and molecular data, and we store these in a graph-based database, creating an internal network. We then look up mutated proteins within the networks and search for related information in the external knowledge sources. This way we build the new general relations network which is considered, finally performing the vertical data integration. We store these enriched relations in the graph-based database, together with the internal relationships

The network has two layers. First layer, covering internal relationships, is built with raw data, i.e. clinical information, expression data, and copy number variants. These are transformed into relationships between patients and proteins. The second layer includes semantically linked data from external domain knowledge sources. These sources provide information about additional proteins related to those existing in our dataset. These new relations are stored in our graph-based database. In order to utilize the additional information from the external knowledge sources we link them within our network via hyperlinks (URLs). This way we can avoid a visual incomprehensibility that would be caused by the redundancy of information. These two layers are combined into one network, where each relation is weighted. Our approach to data integration consists of the following steps (Fig. 3).

All the data from the experimental datasets are integrated horizontally with NoSQL (MongoDB) technology and represented as a semi-structure. This results in a semi-structure per data type, i.e. all clinical data are united in a semi-structure, all expression data in another semi-structure, and all copy number data in a semi-structure. All the raw and metadata are stored in MongoDB in JSON format. In order to integrate the data further, vertically, we first need to find relations between already built semi-structures for clinical records, expression profiles and copy number data. These relationships are managed in the graph-based database − Neo4j. For example, patient A with semi-structure {ID, [attributes]} is related to patient B with semi-structure {ID, [attributes]}. In this relation ID is the important key, while the attributes provide general information about the type of data record (clinical, expression, copy number). Such relations facilitate building a network, different for each studied patient. This network includes expression profiles, copy number, and the mutated proteins. In this way we can detect and link all patients through a specific set of expressed and mutated proteins.

### Linking external data sources

Through semantic data integration, via https RESTFul endpoints (programming access points) specifically, we are able to find additional relationships between proteins from the external domain knowledge sources (EDKS), like Gene Ontology compendium (GO), UniProt, Ensembl [23, 24, 30]. Through EDKS proteins can be found that are closely related to the ones available in the expression profiles. The strength of relation of proteins is established via a score mechanism [30]. For each protein, before importing it into the graph-based database for vertical integration, we search for related proteins. As a result a list of proteins, containing ‘Hugo symbols’ − protein identifiers, is obtained. We use these ‘Hugo symbols’ to find a semi-structure of proteins in our database. The semi-structure is then used to create relationships between the proteins. Thus, relationships between proteins found in our database are generated based also on data from EDKS.

Usually, the number of relationships generated with help of EDKS is unfeasibly large (over a billion), increasing dimensionality of such data. To account for that, we developed a strategy to continue working only with so-called “trusted relationships”. These “trusted relationships” are found by a scoring mechanism. This scoring mechanism is introduced to rank, i.e. score, the most relevant relations (based on semi-structures) originating from our datasets. Internal relationships, based on raw data, have higher score than the relations derived from linked data. We, furthermore, define them as trusted relationships when they occur more than 10 times among different patients. This is necessary for differentiating the significant links between the proteins and for reducing the noise of the relationships between the patients through the added protein information. The noise is introduced by the external knowledge sources, where, potentially, all proteins can be related. The scoring mechanism also ranks the relations originating from external knowledge sources. Naturally, these should have lower scores, compared to the ones derived from the real datasets. In the process of scoring we can also improve the scores of the relations stemming from the external domain knowledge sources on the basis of the frequency with which the certain relation appears. In the next step we classify the already integrated datasets by tumor-related properties. Specifically, we normalize the data by removing the mean and scaling to unit variance [20]. After that, a k-neighbours classification mechanism is applied to split the data into relatively equal groups. Classified data are further used to remove redundant records of the analysed patients. We then normalize the data again by removing the mean and scaling to unit variance.

### Novel integrated tumor-specific feature

For survival time prediction in breast cancer the Nottingham prognostic index (NPI) is usually applied. It helps to determine prognosis following the surgery. Its value is calculated using three pathological criteria: the size of the lesion, the number of involved lymph nodes, and the grade of the tumor. The NPI can be used to stratify patients into groups and is used to predict five-year survival (in accordance with the more commonly used time scales for survival in other types of cancers) [31]. We do not utilize NPI in our framework because it only applies to one specific disease – breast cancer. In our case a universal predictor is essential, in order to account for other cancers, e.g., neuroblastoma. Thus, we develop a novel and universal predictive parameter – Tumor Integrated Clinical Feature (TICF). To predict patient survival time (in both cancer studies combined) we select specific informative clinical features. We tested different features, their combinations and order, and established the optimal setup (not shown). Specifically, the TICF feature is built by numerically concatenating tumor stage, tumor size and age at diagnosis (Fig. 3) in this exact order. The order of concatenation of the clinical data also shows the importance of clinical information for tumor development and relevance to the patient survival rate. A patient with a tumor in stage four, naturally, will have a shorter survival time compared to patients with a tumor in stage two. The next feature – tumor size, is added second because with an increase of the tumor size the survival rate of a patient is reduced. It is also less important to the survival time than the stage of the tumor. Age at the time of diagnosis, is concatenated third, and indicates that older patients have a lower survival rate. If the order of concatenation of these TICF-composing features would differ patients with distant survival-related features would be incorrectly grouped. In this manner, we provide a normalized distance between patients, essential in our subsequent machine learning approaches to survival time prediction.

### Classification and data enrichment

We normalize the TICF feature by subtracting the mean and scaling it according to the unit variance. Centering and scaling are done independently for each record by computing the relevant statistics on the samples. Mean and standard deviation are then stored to be used in later data analysis with the transform method. Patients are then stratified into groups with regard to the TICF similarity using a k-neighborhood approach.

**Fig. 4:**
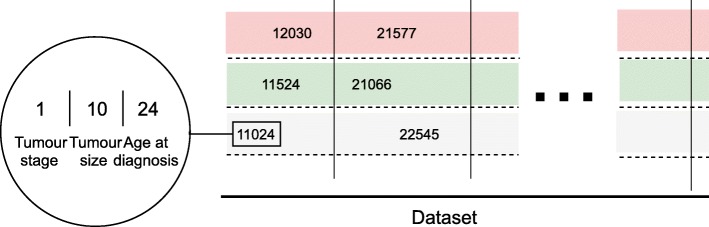
The universal integrated TICF feature. TICF consists of three concatenated initial clinical features: tumor stage, tumor size and age at diagnosis. The columns virtually group the patients by TICF, with regard to the first number – the tumor stage. The rows (split by dotted lines) sort patients according to the values of the TICF, referring to the tumor size and age at diagnosis – always from left to right, following the growth of numerical axis

Using the TICF we find a group of patients most relevant to and build an individual dataset for every studied patient (Fig. 4). In the first step this dataset contains only patients from the found group. It contains information about the TICF and the related mutated proteins. In the already semantically integrated datasets we search for other relations between mutated proteins and patients. These relations can be found within the vertically integrated data. Within each of the defined patient groups we detect relations of these patients to certain proteins. Using these proteins we find relations to other patients, who have the same mutated proteins as in the selected group. These relations are all based on internal relationships. We, thus, enrich each defined group with new related patient records. The next step is to extend the number of related proteins of the selected group by using linked data, based on external knowledge sources. We, again, enrich the defined group of patients through new relations to proteins, and then to other related patients. To avoid redundancy of the linked data relationships we apply the scoring mechanism. This generates a massive dataset, which is different for each patient.

### Survival time prediction models

Next we apply machine learning models to predict and validate survival time of the patients. Artificial intelligence, and in particular machine learning models, has been regularly used in cancer research, with practical implementations [32]. Artificial neural networks and decision trees, for example, have been used in cancer detection and diagnosis for nearly 30 years [33]. Various models, applying Support Vector Machine (SVM) to cancer prognosis, have been successfully used for approximately two decades [34].

Machine learning models used in our study are based on Support Vector Regression (SVR) with different kernels: Radial Basis Function (RBF), Linear and Poly, and Decision Tree Regression model (DTR). Similar models were shown to perform well for survival prediction in cancer studies [35, 36]. Moreover, using these models facilitates a seamless cross-validation.

The TICF features are built for a selected group of patients. We extend the selected group of patients with new closer patients from internal networks and linked data. This newly built set of patients includes enriched TICF features. This set of already enriched TICF features for the selected group and respective relations are used as an input – first parameter to the machine learning models. Second parameter is a number which represents a patient’s survivability. For the survivability prediction model we use the count of months after cancer is diagnosed, information available for most of the patients in studied datasets. As a result, the machine learning models return an approximate value which represents survival time in count of months. As mentioned, these first models are based on Support Vector Regression with different kernels. SVR with RBF can be defined as a simple single-layer type of an artificial neural network called an RBF network. This RBF is used as an interpolation approach which ensures that the fitting set covers the entire data equidistantly. SVR-Linear represents a function for transforming the data into a higher dimensional feature space in order to enable a linear separation. SVR-Poly represents the similarity of vectors (training samples) in a feature space over polynomials of the original variables, allowing learning of non-linear models. The feature space of a polynomial kernel is equivalent to that of polynomial regression. In the SVR-DTR, a decision tree represents a regression or classification model in the form of a tree structure. It breaks down a dataset into smaller subsets while at the same time an associated decision tree is incrementally developed. The final result is a tree with decision nodes and leaf nodes.

These models fit the features selected for survival time prediction from our integrated dataset and the yielded results are directly comparable.

### (Cross-)validation

We validate the outcomes of the applied machine learning models by using randomly smaller subsets of both raw and integrated data, in a cross-validation setup. Specifically, a k-fold cross-validation is applied, where the original sample is randomly partitioned into k equal-size subsamples. Of the k subsamples, a single subsample is retained as the validation data for testing the model, and the remaining k −1 subsamples are used as training data. The cross-validation process is then repeated k times (the folds), with each of the k subsamples used exactly once as the validation data. The k results from the folds can then be averaged to produce a single estimation. The advantage of this method over repeated random sub-sampling is that all observations are used for both training and validation, and each observation is used for validation exactly once. 10-fold cross-validation is commonly used, but in general, k remains an unfixed parameter. This validation model can be used to estimate any quantitative measure that is appropriate for the data and the model.

## Results

### Semantic data network

We developed a novel network-based data integration model, where we combine clinical and molecular data, using both raw data records and external knowledge sources. Relations derived from the raw data represent the internal network and relations based on external domain knowledge sources (EDKS) are represented as a semantically linked network. Our semantically linked network is connected to EDKS via RESTFul API endpoints. These endpoints are different for each type of EDKS. As a result we use two types of EDKS data. The first type consists of proteins from GO, related to the studied protein, based on scores provided in the GO. The second type of data we use, includes additional information about proteins in the raw data, e.g., ‘Hugo Symbol’. These proteins often are not completely defined by families and domains, so we use the Hugo symbols and search for these protein domains and families through the EKDS. Using similar proteins from EDKS (GO) we semantically enrich our internal network with new knowledge about relations between proteins, which cannot be derived from the raw data. The resulting highly dimensional network, consisting of more than one billion relations, includes redundant information, which we reduce via our scoring mechanism. Technically, the fusion of the two studied types of cancer involves both horizontal and vertical data integration, using two different database models. The first is a document database model where all heterogeneous raw data are integrated. The second is a graph database model where all different types of relations between patients and proteins are joined. For the purpose of survival time prediction, combining clinical information, we developed a novel universal Tumor Integrated Clinical Feature (TICF). The TICF features are first identified using the raw data, based on three existing clinical features – tumor stage, tumor size and age at diagnosis. The TICF features are then used to create patient similarity network that, in the next step, is further extended with molecular information.

**Fig. 5:**
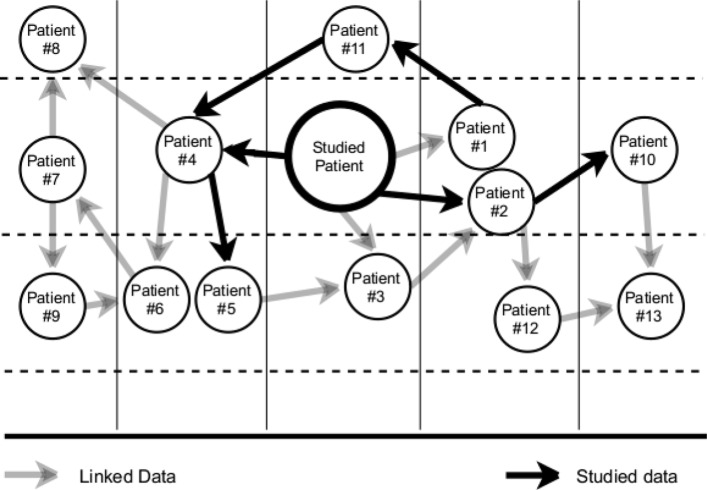
Example of patients related semantically via internal and linked network. The studied patient of interest is shown as a larger circle in the middle. The vertical lines split the schema into 5 classes, as determined by the k-neighbor classification of the TICF feature. Black arrows show patients related to the studied patient based on the molecular information. Grey arrows distinguish patients related to the studied patient based on external linked data (from EDKS)

Figure 5 shows an example of a network of patients that are semantically related to a studied patient – patient we are interested in analysing. We build a TICF for all the patients for whom the necessary clinical information is available. Focusing now on a patient of interest, we then find patients related to her by TICF similarity. Specifically, we use the k-neighbours model to split patients into 5 classes. This initial group contains a small set of patients because not every patient has a TICF feature. In the subsequent steps of the study, we use the molecular data and find all proteins related to this selected group of patients. Considering relationships between these proteins, based on the molecular data, we can link patients with each other. Next, we find all semantically related proteins using the external, i.e. linked, data (EDKS) to still find additional related patients. We can then combine all information – internal and linked relations, from the found group into one new extended, semantically enriched dataset.

Machine learning models for survival time prediction can then be applied to any patient within this dataset who has a defined TICF feature.

### Machine learning models for survival time prediction

Groups obtained via the TICF feature, naturally, can be unbalanced. For example, including patients with smaller number of data records – which presents an obstacle for predicting the survival time. For validation we focus on smaller datasets (approximately 25% of the whole dataset) from the raw data which are clustered into 5 subgroups by using the k-fold algorithm.

After the dataset is normalised and patients stratified into groups we apply several machine learning models for survival time prediction: Support Vector Regression (SVR with RBF, Linear and Polynomial kernels) as well as Decision Tree Regression (DTR).

**Fig. 6:**
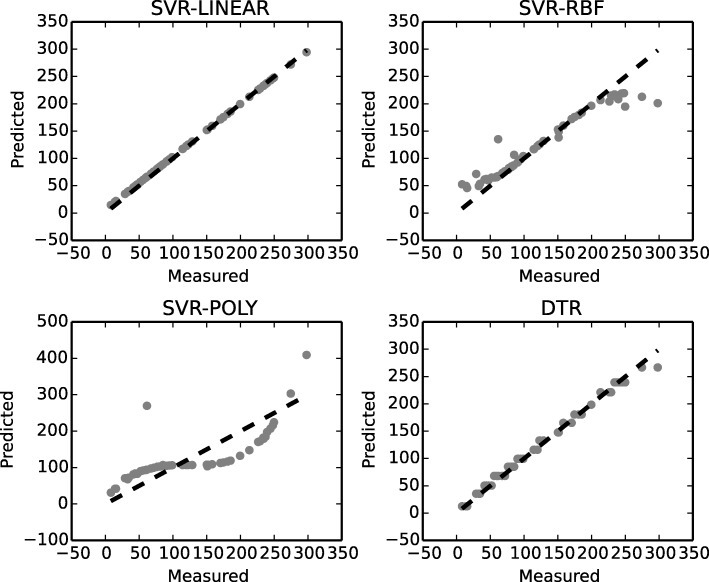
Success rates of machine learning models for survival time prediction. Predicted-to-measured values of survival time prediction (by TICF), where numbers correspond to months of survival time prediction. A dotted line symbolizes an ideal case of the ratio predicted to measured TICF for survival time prediction

In Fig. 6 performance – accuracy, of the machine learning models applied to survival time prediction is shown. Survival time is predicted using the data of both cancers combined and with our framework used for processing and integrating the data. Decision Tree Regression (DTR) and Support Vector Regression with linear kernel (SVR-Linear) perform best, the latter yielding the most accurate results for survival time prediction. The potential of these models is in improving the accuracy of survival time prediction by improving iteratively the training dataset over the whole integrated dataset. Specifically, with every new studied patient we iterate over, we enrich the training dataset with new trusted relations from our linked relationships, defined by the increased frequency of their use.

**Fig. 7:**
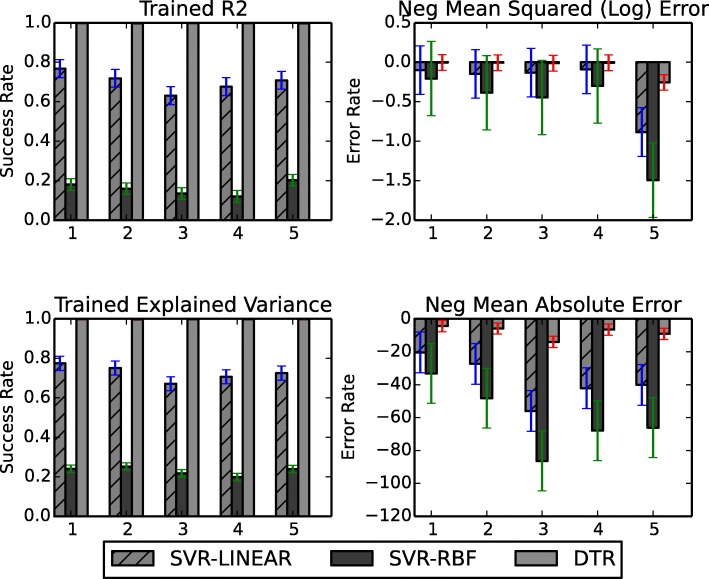
Cross-validation of the applied machine learning models for survival time prediction. Performance of three models (SVR-Linear, SVR-RBF and DTR) is compared. The fourth model, SVR-Poly has shown biased results (Fig. 6). Cross-validation is based on 5 subgroups defined by the TICF feature, obtained after using k-fold algorithm. On the vertical axis the mean values with standard deviations of success rates and error rates of the matchings of TICF feature are given

Next we compare the applied models in a cross-validation approach (Tab. 1). The validation is based on four parameters for error evaluation: trained R2 (coefficient of determination) and trained explained variance are related to the accuracy of the used model; while trained negative mean square log error and negative mean absolute error are related to the noise (error) level. The resulting accuracies (Fig. 7) again confirm that the SVR-Linear and DTR models using TICF outperform other models, i.e. SVR-RBF, with regard to accuracy. This shows that SVR-Linear and DTR are more suitable, among the four compared models, for accurate survival time prediction.

**Table 1:** Aggregated results of cross-validation

ML Model	Train R2		Explained Variance		Negative Mean		Negative Median	
					Absolute Error		Absolute Error	
	Mean	StD	Mean	StD	Mean	StD	Mean	StD
SVR-RBF	0.318	0.038	0.341	0.032	−45.742	5.224	−34.455	5.594
SVR-LINEAR	0.983	0.007	0.986	0.006	−7.288	1.935	−6.109	1.509
DTR	0.996	0.000	0.996	0.427	−5.624	0.427	−4.636	0.467
SVR-POLY	0.884	0.007	0.887	0.009	−20.354	3.290	−15.581	5.382

To confirm that our integrative framework is indeed necessary to obtain best performance, we examine alternative approaches (see the Additional files 1, 2, 3 and 4). First we show cross-validation results using our relational network but without the TICF integrated clinical feature (Additional file 1). Instead we use the clinical features as separate regressors in the experiment. Next we use the TICF feature but this time we do not extend the patient similarity search with the relational network (Additional file 2). Finally, we look at predictions when only separate clinical features are used and no relational network (Additional file 3). Executional times of the applied ML models are given in Additional file 4. As is evidenced, models building on our novel fully integrative framework outperform the alternatives.

## Discussion

In this work we introduce a novel unified and universal approach for integration of data generated in independent cancer studies. We demonstrate its application to breast cancer and neuroblastoma datasets. Our model is built to facilitate application and extension to multiple different diseases with different types of multi-omics data. Subsequently, we highlight clinical relevance of our data integration method by applying it to survival time prediction, using machine learning models.

The original contribution of our work is the data integration model. A number of interesting and different approaches, related to the similar problem were presented in the previous CAMDA challenges [26]. We developed our strategy for data integration by using a semantically defined network approach based on different database models. Major objectives in our integrative framework are to integrate and utilize information, also latent, available in whole and dynamically growing datasets for multiple diseases. Additionally to the potential extensibility of our data integration model, it also facilitates a seamless integration with external knowledge sources. The data integration challenge was solved by using models for horizontal and for vertical integration. Specifically, we applied new database technologies: document type database for horizontal integration and graph database for vertical integration – MongoDB and Neo4j, respectively. Such software technology facilitates finding relations between the records in the integrated datasets. The main merit of our approach is that we are able, also dynamically, to add more data and relations. We explore these opportunities by adding new semantically defined relations from the external knowledge sources. Such approach gives us not only a solution to the particular task of the CAMDA challenge, but can also be applied in similar research and practical projects. Our software platform can be easily extended and supported.

Moreover, we apply and compare the performance of multiple machine learning models that use the semantically linked data. Specifically, we develop a new classification feature for survival time prediction.

The new feature – TICF, is an integrated parameter, allowing semantical enrichment through the semi-structure generated by our novel data integration model. TICF can be used for the application of certain machine learning models in order to find patients closely related to the studied one. Inclusion of related patients with different clinical and expression parameters, as we show, is essential for improving the accuracy of survival prediction models.

For survival time prediction we apply supervised regression models [35, 36]. Models used in this study utilize the TICF feature to improve the accuracy of patient survival time prediction. Moreover, application of these specific machine learning algorithms ensures a reliable validation of our semantic data integration approach. Cross-validation of these models showed stable results with regard to achieved accuracy – both in the context of success and error rates, in survival time prediction.

## Conclusions

We developed models for defining and enriching relations by integrating data of various types and from disparate sources (two different cancer datasets), and consolidating them into meaningful and valuable information by the use of semantic technologies. The use of linked and overlayed NoSQL database technologies allowed us to aggregate the non-structured, heterogeneous cancer data with their various relationships. The applied semantic integration of different cancer datasets facilitates an enrichment of the studied data by discovery of mutual internal relations and relations with external domain knowledge sources.

We developed machine learning based models for survival time prediction in two types of cancer – breast cancer and neuroblastoma. We proposed a novel universal and integrative feature for classification and analysis, investigated its performance in a cross-validation setup with four machine learning models, and showed that the best results are obtained with our integrative framework. Specifically, using Support Vector Regression with Linear kernel, and Decision Tree Regression.

## Reviewers’ comments

### Reviewer’s report 1: Eran Elhaik, Ph.D

The proposed framework is indeed novel but I found the manuscript long and difficult to read. It should be shortened and more figures should be employed to better explain it. This can be done by adding figures showing the pipeline and how the framework works and revise the legends to be more informative. Typos should be corrected. The manuscript is original and significant for works in this field.

Author’s response: *We agree with the suggestions and improved the text. Figures illustrating the pipeline and how the framework works are now adjusted for better clarity. Typos are corrected and the text is optimized.*

### Reviewer’s report 2: Eran Elhaik, Ph.D

Is there a link to the system?

Author’s response: *The system is developed internally and, at the moment, not for public use. However, we uploaded the latest version of the source code into a repository on GitHub, accessible after requesting permission. Our aim is to develop the system as a publicly accessible tool.*

### Reviewer’s report 3: Eran Elhaik, Ph.D

What are the results of the framework that has been applied to the 2 cancers? You wrote: “The potential of these models is in improving the accuracy of survival time prediction by improving iteratively the training dataset over the whole integrated dataset.” Where can we see the improving of the results accuracy?

Author’s response: *The potential of these models is shown in Figs.* 6 *and* 7* and in the now additionally introduced table. The more similar the data between two patients, the more accurate the prediction is. We have now added a table with aggregated numerical results with regard to accuracy, as indicated by R2.*

### Reviewer’s report 4: Eran Elhaik, Ph.D

Figure 4 is unclear.

Author’s response: *In Fig.* 4* we present the idea of TICF and how it is organized.*

### Reviewer’s report 5: Eran Elhaik, Ph.D

Figure 5 is most unhelpful. Consider adding more information from proteins, etc. to increase clarity.

Author’s response: *Fig.* 5* shows how the Linked Data concept is applied for patient data integration. The linked data is a method of publishing structured data so that it can be interlinked and explored via semantic queries. The queries in our case can be done by using the information of the relation between a mutated base (e.g., CNVs) and a patient.*

### Reviewer’s report 6: Eran Elhaik, Ph.D

The legends of Figs. 6 and 7 are unclear. If I understand correctly, Fig. 6a demonstrates the accuracy of the model -this should be emphasizes and more numerical results should be provided, but for which cancer are they? Also, these figures have subplots that are not mentioned in the legend.

Author’s response: *We introduced Tab. *1* showing the numerical results. We give an answer to the rest of the question in our answer to question 3.*

### Reviewer’s report 7: Eran Elhaik, Ph.D

TCIF is used before it is defined.

Author’s response: *Text is now adjusted.*

### Reviewer’s report 8: Eran Elhaik, Ph.D

Page 2, line 6: in order to store, to access, to analyse and to mine it easily. -> to store, access, analyse and mine it easily.

Author’s response: *Text is now adjusted.*

### Reviewer’s report 9: Eran Elhaik, Ph.D

You constantly using analysis when it should have been analyses.

Author’s response: *Text is now adjusted.*

### Reviewer’s report 10: Eran Elhaik, Ph.D

Data are Plural

Author’s response: *Text is now adjusted.*

### Reviewer’s report 11: Eran Elhaik, Ph.D

Abstract: integration were both clinical and molecular ->integration where both clinical and molecular

Author’s response: *Text is now adjusted.*

### Reviewer’s report 12: Eran Elhaik, Ph.D

P. 3, line 18. “Through combining data from multiple cancers in this way we create a network of data where entities, like proteins, clinical features and expression features, are linked with each other” explain now.

Author’s response: *Fig.* 5* shows how the Linked Data concept is applied for patient data integration. The linked data is a method of publishing structured data so that it can be interlinked and explored via semantic queries. The queries in our case can be done by using the information of the relation between a mutated base (e.g., CNVs) and a patient.*

### Reviewer’s report 13: Eran Elhaik, Ph.D

P. 3, line 26-40. There is a need for a figure here, which would show the workflow and use the terms used in this manuscript, like “entity”

Author’s response: *Text is now adjusted.*

### Reviewer’s report 14: Eran Elhaik, Ph.D

P. 4, line 23. “Conventional classification k-neighbours method is used to find patients that are linked most closely to the studied one.”İ Explain how.

Author’s response: *Fig.* 5* shows how the Linked Data concept is applied for patient data integration. The linked data is a method of publishing structured data so that it can be interlinked and explored via semantic queries. The queries in our case can be done by using the information of the relation between a mutated base (e.g., CNVs) and a patient. KNN Method is used to classify patients based on TICF features.*

### Reviewer’s report 15: Eran Elhaik, Ph.D

In Fig. 1, I suggest that you add another figure (ab) that shows the integration of data for 2 patients because this is unclear

Author’s response: *Fig.* 1* could potentially be extended. However, the integration of data between two (respectively all) patients is already shown in Fig.* 5*, where the concept of linked data is presented - the methodological background of our study.*

### Reviewer’s report 16: Eran Elhaik, Ph.D

Section 4 - I suggesting using a figure showing a case study being processed.

Author’s response: *We now added the below text to the Fig.* 3*.: In data preparation phase we transform and store raw data with different formats in a document database which is considered as horizontal data integration. After that we generate the relations between data based on the raw dataset for patients, molecular data and we store them in a graph based database thus we developed the internal network. After that for every mutation we search related information from external knowledge sources and build the new general relations network which is considered as vertical data integration. We store these enriched relations in the graph based database together with the internal relationships.*

### Reviewer’s report 17: Eran Elhaik, Ph.D

P.4. “The first approach, called here ‘internal data network’, requires data to be expressed in terms of internal relations.” give examples to such connections and where connections cannot be found

Author’s response: *Connections are based on the relations of patients to proteins, information directly available in the raw data. However, there exist no data concerning relations between proteins and these cannot be derived from the internal data network. We discover these protein relations based on the linked data, finally finding the relations between patients.*

### Reviewer’s report 18: Eran Elhaik, Ph.D

P. 7. Line 29. “These trusted relationships” are found by a scoring mechanism. This scoring mechanism is introduced to rank, i.e. score, the most relevant relations (based on semi-structures) originating from our datasets." Can you expand on that?

Author’s response: *Internal relationships, based on raw data, have higher score than the relations derived from linked data. We, furthermore, define them as trusted relationships when they occur more than 10 times among different patients. This is necessary for differentiating the significant links between the proteins and for reducing the noise of the relationships between the patients through the added protein information. The noise comes from external knowledge sources where potentially all proteins can be related.*

### Reviewer’s report 19: Eran Elhaik, Ph.D

P. 7. Line 39. “In this way we normalize the data.” Unclear how.

Author’s response: *Specifically, we normalize the data by removing the mean and scaling to unit variance [*20*]. After that a classification mechanism is applied to split the data into relatively equal groups.*

### Reviewer’s report 20: Eran Elhaik, Ph.D

P 10, line 58. “took some smaller datasets from the raw data” how small?

Author’s response: *It is 25%. We added this information in the text.*

### Reviewer’s report 21: Eran Elhaik, Ph.D

Check reference 4. “Bio*Medical Informatics”? reference 12 has a lot of authors. Reference 20 what’s going on there? Check all your references.

Author’s response: *Adjusted in the text now.*

### Reviewer’s report 1: Wenzhong Xiao

While the overall framework of the work is described in the text and the figures and can be understood conceptually, the details of the approach applied to the CAMDA datasets of the two cancers are apparently lacking or difficult to follow at places, making it difficult to evaluate the quality of the resulting network after the integration. For examples, a) on page 6 line 61, “By generating such relations a network is built, different for each studied patient. This network includes expression profiles, CNV for the horizontal integration, and the mutated proteins.” How does this network look like for a patient? What are the different types of nodes and relationships on the network? (optional) Just as a suggestion, the clarity of these details can potentially be easily addressed by a figure showing a representative small region of the network of a selected patient. b) on page 7, line 27, “we developed a strategy to continue working only with so-called trusted relationships... by a scoring mechanism.” What was the scoring mechanism used?

Author’s response: *a) We agree with the remark and we think the graphical interpretation of the reply can be seen in Fig.* 5*, which is the methodologic background of our study. b) Internal relationships, based on raw data, have higher score than the relations derived from linked data. W, furthermore, define them as trusted relationships when they occur more than 10 times among different patients. This is necessary for differentiating the significant links between the proteins and for reducing the noise of the relationships between the patients through the proteins. The noise comes from external knowledge sources where potentially all proteins can be related.*

### Reviewer’s report 2: Wenzhong Xiao

The “integrated tumor specific feature” proposed by the authors (tumor size, tumor stage, and age at the time of diagnosis) across different cancers is innovative and potentially important. Again, some of the details are apparently missing. For examples, a) on page 8, line 41, “Within each of the defined patient groups we detect relations of these patients to certain proteins. Using these proteins we find relations to other patients, who have the same mutated proteins as in the selected group.”İ What was the algorithm used? This is particularly relevant since it is not clear to this reviewer how the mutated proteins are identified for each patient in the two datasets, as apparently there is no DNA sequencing data in ether of the datasets b) similarly, on page 8, line 47, “The next step is to extend the number of related proteins of the selected group by using linked data, based on external knowledge sources. We again enrich the defined group of patients through new relations to proteins, and then to other related patients.” Again, what was the algorithm? c) on page 10, line 34, is there a difference in “weight”İ between the connection of the patients based on studied data and linked data? If yes, how the weights were defined? (optional) Just as a suggestion, could the author please consider demonstrating any improvement in performance by integrating the datasets of the two cancers comparing with only the data from one cancer?

Author’s response: *a) Molecular raw data consist of records for each patient - their copy number information (CNV) and expression profiles, where each value is reported per protein (as a unique Hugo Symbol). Based on that we develop relations between patients and proteins, i.e. the internal relationships creation. b) The names, i.e. Hugo Symbols, of the mutated proteins are obtained from the raw datasets directly. A particular Hugo Symbol is searched for in a specific external knowledge source, e.g., in Uniprot, considering also related Hugo Symbols. This way, through vertical integration, external network is developed by relating the proteins to patients, i.e. patient records. This is the conceptual background for our study. c) Internal relationships, based on raw data, have higher score than the relations derived from linked data. W, furthermore, define them as trusted relationships when they occur more than 10 times among different patients. This is necessary for differentiating the significant links between the proteins and for reducing the noise of the relationships between the patients through the proteins. The noise comes from external knowledge sources where potentially all proteins can be related.*

### Reviewer’s report 3: Wenzhong Xiao

On page 1, line 23, “With lack of effective integration strategies novel models are necessary for efficient and operative data integration were both clinical and molecular information can be effectively combined.” “were” should be “where”.

Author’s response: *Adjusted in the text now.*

### Reviewer’s report 4: Wenzhong Xiao

On page 1, in the Abstract, consider keeping the verb tenses consistent.

Author’s response: *Adjusted in the text now.*

### Reviewer’s report 5: Wenzhong Xiao

In Fig. 2, how do the authors extract mutation information from the aCGH and Illumina gene profiles?

Author’s response: *For the aCGH we use properties, i.e fields (columns), “sample_characteristics_CH1” or “sample_characteristics_CH2” and other to define the relations of the tumors with expression profiles of the patients from where we can relate the genes and the mutations respectively. For Illumina gene profiles the approach is similar.*

### Reviewer’s report 6: Wenzhong Xiao

In Fig. 3, consider including aCGH, Affymetrix SNP6.0 and patient information (which are shown in Fig. 2) in this flowchart

Author’s response: *We now edited Fig.* 3* for better clarity.*

### Reviewer’s report 1: Carlos Loucera

The authors propose an interesting approach to data integration based on the construction of a relational network that combines heterogeneous data, like different views of the each sample, expression of distinct tumors or patterns mined from external resources. Furthermore, the authors propose a novel score (TICF) in order to predict the survival time of a patient. This score is defined by combining several categorical variables into a single ordinal characteristic. Finally, the survival prediction model is validated using a classical k-fold cross-validation strategy. My main criticism to the paper is that the authors do not provide enough information on the different methods and algorithms that make up the proposed methodology. It is very difficult to implement the paper as it is, even if the data is available (by accepting CAMDA terms). Regarding the data, there is a lack of descriptive statistics and contextual information. The text is written by a person who has worked with the CAMDA datasets in mind, which in my humble opinion is a mistake.

Author’s response: *We think that it is important for the research community to be able to test and even extend software presented in scientific publications. Therefore, we uploaded the latest version of the source code of our tool into a repository on GitHub, accessible after requesting permission. Our ultimate goal is to develop the system as a publicly accessible tool. Importantly, our aim for the moment is to present our approach and to discuss its potential applications. We are, moreover, actively working on extending the tool to other datasets. With regard to the datasets, as they had been previously thoroughly characterised in the corresponding publications (METABRIC: Curtis et al., Nature 486, and Dream Challenge, Margolin et al, Sci Transl Med 5; Neuroblastoma: Fischer lab, KÃűln - Stigliani et al, Neoplasia 14, Coco et al, IJC 131, Kocak et al, Cell Death Dis 4, Theissen et al, Genes Chromosomes Cancer 53), we decided to not go into details for the sake of space. We do, however, briefly present the data in the *‘Data description’* section of the *‘Material and methods’.

### Reviewer’s report 2: Carlos Loucera

Although the methodology provides an interesting framework to combine several information sources into a single structured model, a relational network, the authors do not provide any kind of biological or clinical interpretation: neither the mined relations nor the results. I do not understand how the TICF features are used.

Author’s response: *We show biological relevance of our results by using the survival analysis as the example use case. We describe how the TICF features are used in Fig.* 4.

### Reviewer’s report 3: Carlos Loucera

In page 9, line 14 the authors refer to an enriched TICF for a selected group of patients. This arises the first question, how is this group selected? Later, in line 17, the author refers to the second parameter (the survival score) in a per patient basis. As I understand it, the group enriches the TICF feature. How? What are the main differences between the combined feature and the enriched one? These questions should be addressed in the paper. The authors should clarify this essential part of the methodology since it seems a dangerous loop: group by TICF, extract knowledge and feature enrichment of TICF.

Author’s response: *Corrections of the text on page 9, line 14: “(...) The TICF features are built for a selected group of patients. We extend the selected group of patients with new closer patients from internal networks and linked data. This newly built set of patients includes enriched TICF features. This set of already enriched TICF features for the selected group and respective relations are used as an input - first parameter to the machine learning models.”*İ

### Reviewer’s report 4: Carlos Loucera

From a machine learning point of view the paper lacks in several aspects. First and foremost, a score is used to reduce the network size, how this score is constructed should be well documented in the paper, which it is not the case. There are two critical unanswered questions, what kind of distribution the score has and how the decision threshold is computed.

Author’s response: *We thank for this very valuable remark and address the concerns below. Internal relationships, based directly on raw data, have higher score than relations derived from the linked data. We, furthermore, define them as trusted relationships when they occur more than 10 times among different patients. This is necessary for differentiating significant links between the proteins and for reducing the noise of the relationships between the patients through the proteins. The noise comes from external knowledge sources, where potentially all proteins can be related.*

### Reviewer’s report 5: Carlos Loucera

Although the problem at hand deals with survival prediction, the authors only provide a classical regression solution. In regard to the standardization of the TICF feature, the implementation rises many questions. As outlined in the paper the parameters are learned using the whole dataset (lines 26-33) but it should be learned in a per-fold basis: learning the preprocessing parameters during the training phase, then applying the learned transformation to the validation subset. Furthermore, the estimator comparison is not well constructed, from the regression scores it seems that the hyperparameters for the RBF and Polynomial kernels are not optimized for the dataset.

Author’s response: *We start training the models while the data are integrated and the TICF features generated. This is an initial training and for each studied patient the models are additionally trained for each new patient by finding new relations. In general, survival time prediction is only used as an example application of the framework. For survival time prediction in both cancers the TICF feature is used for classification purposes using the KNN model. After the dataset is normalised and patients stratified into groups we apply several machine learning models for survival time prediction: Support Vector Regression (SVR with RBF, Linear and Poly kernels) as well as Decision Tree Regression (DTR).*

### Reviewer’s report 6: Carlos Loucera

This leads to a very important question for which the authors do not provide any answer: how the hyperparameters are set. To be more confident about the validation I would expect a nested cross-validation scheme. As the TICF feature is used by combining other features and later enriched, I would expect a performance comparison between the three sets of features: the not-combined ones, the combined but unenriched ones and the combined and enriched cases. In its current form the paper feels like an extended abstract, promising but unfocused.

Author’s response: *This is a very valuable suggestion. We here want to first show and discuss the potential of vertical and horizontal data integration on cancer data use cases. Statistical testing of the features is not the major scope of this first publication.*

### Reviewer’s report 7: Carlos Loucera

The validation rises many questions, why do the authors only use a subset of the data?. The method is not properly explained to the point that it could be very hard to implement it,not to mention the inability to reproduce the results. Due to these facts I do not endorse the publication of the paper in its current form.

Author’s response: *In our opinion, the validation models are applicable and comprehensive. For the sake of reproducibility, we now uploaded the latest version of the source code into a repository on GitHub, accessible after requesting permission. We also reproduce the results in an extended study (see: **https://www.mdpi.com/2078-2489/10/3/93*).

### Reviewer’s report 8: Carlos Loucera

- Since scikit-learn has been used, in order to deal with the survival analysis problem I highly recommend either the scikit-survival or the lifelines python package. - I recommend the authors to find the best hyperparameters and perform the validation by means of a nested cross-validation scheme. The scikit-learn package has a well documented example. - Maybe the authors are using the default hyperparameter values provided by the scikit-learn API? If so, I recommend to implement a hyperparameter search strategy (even a simple random search could yield better results for the RBF and Polynomial kernels) - I recommend the authors to extract some kind of interpretation of the constructed network with some sort of unsupervised learning algorithm working on the graph. - I highly encourage the authors to better describe the methodology, be more careful about the validation schema and use the whole dataset when dealing with the validation. - I would include some stratification of the validation metrics, such as stratifying the results by tumor type and subtype (if available).

Author’s response: *We thank the reviewer for the valuable recommendations. We did address some of them already in our answers to previous questions. Our aim is to develop a tool based on the methodologies we used in the study, and the tool will consider your recommendations.*

### Reviewer’s report 9: Carlos Loucera

Poly kernel sounds too informal, use polynomial kernel instead

Author’s response: *Adjusted in the text now.*

### Reviewer’s report 10: Carlos Loucera

It is often advised to perform a simple random split (with a fixed set of hyperparameters) of the data and analyze the results on the test set (in addition to other validation schemes, in order to showcase a typical use case).

Author’s response: *We are grateful to the reviewer for his suggestion and we will consider it in the extension of our study.*

### Reviewer’s report 11: Carlos Loucera

When talking about the first and second parameters (lines 14-16, page 9), you should use more standard names, such as features (input) and response (output).

Author’s response: *We agree with the suggestion.*

### Reviewer’s report 12: Carlos Loucera

Once all the previous issues have been resolved, I would recommend to revise the English, although it should be mentioned that the text is concisely written, in an easy to follow way

Author’s response: *We now revised the text.*

## Supplementary information

**Additional file 1** **Table S1**. Aggregated results of cross-validation, using separate clinical features, i.e. no TICF; and relational network.

**Additional file 2** **Table S2**. Aggregated results of cross-validation, using TICF; without the relational network.

**Additional file 3** **Table S3**. Aggregated results of cross-validation, using separate clinical features, i.e. no TICF; without the relational network. NA means that the model didn’t converge.

**Additional file 4** **Table S4**. Execution time of the applied ML models per iteration. in terms of time of training, predict and total time which are sum of the train plus predict time.

## Data Availability

All scripts are provided in git repositories and are available upon request.

## Abbreviations

API: Application programming interface
BC: Breast cancer
CAMDA: Critical assessment of massive data analysis
CD: Coefficient of determination
CNV: Copy number variance
DTR: Decision tree regression
EDKS: External domain knowledge sources
GO: Gene ontology compendium
HTTP: Hypertext transfer protocol
JSON: JavaScript object notation
NB: Neuroblastoma
NoSQL: Not only structured query language - non relational database
NPI: Nottingham prognostic index
RBF: Radial basis function
RESTful: Representational state transfer
RNA-seq: RNA sequencing
SVM: Support vector machine
SVR: Support vector regression
TICF: Tumor integrated clinical feature
URIs: Uniform resource identifiers
W3C: World wide web consortium
