# Supplementary material for: A novel framework for horizontal and vertical data integration in cancer studies with application to survival time prediction models
Source: Biol Direct. 2019 Nov 21;14:22. doi: 10.1186/s13062-019-0249-6 (PMC6868770; doi:10.1186/s13062-019-0249-6)
Supplement: Supplementary file 1 — Additional file 1 Table S1. Aggregated results of cross-validation, using separate clinical features, i.e. no TICF; and relational network. [file 13062_2019_249_MOESM1_ESM.pdf]

Table A. Aggregated results of cross-validation, using separate clinical features, i.e. no TICF; and relational network.

ML Model	Train R2		Explained Variance		Negative Mean Absolute Error		Negative Median Absolute Error	
	**Mean**	**StD**	**Mean**	**StD**	**Mean**	**StD**	**Mean**	**StD**
SVR-RBF	0.206	0.044	0.230	0.023	−54.923-	5.267	−49.310	8.098
SVR-LINEAR	0.972	0.009	0.973	0.008	−5.494	1.009	−5.312	1.515
DTR	0.991	0.004	0.991	0.004	−5.258	1.125	−4.443	1.235
SVR-POLY	0.883	0.020	0.886	0.021	−18.900	1.112	−14.272	3.289
