# Supplementary material for: A novel framework for horizontal and vertical data integration in cancer studies with application to survival time prediction models
Source: Biol Direct. 2019 Nov 21;14:22. doi: 10.1186/s13062-019-0249-6 (PMC6868770; doi:10.1186/s13062-019-0249-6)
Supplement: Supplementary file 2 — Additional file 2 Table S2. Aggregated results of cross-validation, using TICF; without the relational network. [file 13062_2019_249_MOESM2_ESM.pdf]

**Table B. Aggregated results of cross-validation, using TICF; without the relational network.**

| ML Model | Train R2 | | Explained Variance | | Negative Mean Absolute Error | | Negative Median Absolute Error | |
|---|---|---|---|---|---|---|---|---|
| | **Mean** | **StD** | **Mean** | **StD** | **Mean** | **StD** | **Mean** | **StD** |
| SVR-RBF | 0.119 | 0.171 | 0.283 | 0.041 | −35.024 | 3.852 | −31.768 | 8.111 |
| SVR-LINEAR | 0.876 | 0.024 | 0.896 | 0.015 | −13.733 | 2.758 | −12.726 | 2.579 |
| DTR | 0.984 | 0.011 | 0.988 | 0.006 | −4.727 | 1.760 | −4.449 | 1.709 |
| SVR-POLY | 0.006 | 1.845 | 0.102 | 1.708 | −21.943 | 13.774 | −14.637 | 4.944 |
