# Supplementary material for: A novel framework for horizontal and vertical data integration in cancer studies with application to survival time prediction models
Source: Biol Direct. 2019 Nov 21;14:22. doi: 10.1186/s13062-019-0249-6 (PMC6868770; doi:10.1186/s13062-019-0249-6)
Supplement: Supplementary file 3 — Additional file 3 Table S3. Aggregated results of cross-validation, using separate clinical features, i.e. no TICF; without the relational network. NA means that the model didn’t converge. [file 13062_2019_249_MOESM3_ESM.pdf]

Table C. Aggregated results of cross-validation, using separate clinical features, i.e. no TICF; without the relational network. NA means that the model didn't converge.

ML Model	Train R2		Explained Variance		Negative Mean Absolute Error		Negative Median Absolute Error	
	**Mean**	**StD**	**Mean**	**StD**	**Mean**	**StD**	**Mean**	**StD**
SVR-RBF	*NA*	*NA*	*NA*	*NA*	*NA*	*NA*	*NA*	*NA*
SVR-LINEAR	0.866	0.025	0.877	0.029	−14.485	2.359	−12.594	2.025
DTR	0.985	0.013	0.987	0.011	−4.526	2.133	−4.088	1.684
SVR-POLY	0.764	0.073	0.805	0.033	−17.165	1.376	−14.516	1.963
