# Supplementary material for: A novel framework for horizontal and vertical data integration in cancer studies with application to survival time prediction models
Source: Biol Direct. 2019 Nov 21;14:22. doi: 10.1186/s13062-019-0249-6 (PMC6868770; doi:10.1186/s13062-019-0249-6)
Supplement: Supplementary file 4 — Additional file 4 Table S4. Execution time of the applied ML models per iteration. in terms of time of training, predict and total time which are sum of the train plus predict time. [file 13062_2019_249_MOESM4_ESM.pdf]

Table D. Execution time of the applied ML models per iteration. in terms of time of training, predict and total time which are sum of the train plus predict time.

ML Model	Train (s)	Predict (s)	Total (s)
SVR-RBF	0.070589066	0.001976013	0.072565079
SVR-LINEAR	0.050335884	0.000173092	0.050508976
DTR	0.028145075	0.000221968	0.028367043
SVR-POLY	0.479592085	0.001157999	0.480750084
